# Sickle cell disease, sickle trait and the risk for venous thromboembolism: a systematic review and meta-analysis

**DOI:** 10.1186/s12959-018-0179-z

**Published:** 2018-10-04

**Authors:** Jean Jacques Noubiap, Mazou N. Temgoua, Ronni Tankeu, Joel Noutakdie Tochie, Ambroise Wonkam, Jean Joël Bigna

**Affiliations:** 1 Department of Medicine, Groote Schuur Hospital and University of Cape Town, Cape Town, 7925 South Africa; 2 Department of Internal Medicine and sub-Specialties, Faculty of Medicine and Biomedical Sciences, Yaoundé, Cameroon; 3 Department of Surgery and sub-Specialties, Faculty of Medicine and Biomedical Sciences, Yaoundé, Cameroon; 4 Division of Human Genetics, Faculty of Health Sciences, University of Cape Town, Cape Town, South Africa; 5 Department of Epidemiology and Public Health, Centre Pasteur of Cameroon, Yaoundé, Cameroon; 6 Faculty of Medicine, University of Paris Sud XI, Le Kremlin Bicêtre, France

**Keywords:** Sickle cell disease, Sickle cell anemia, Sickle cell trait, Venous thromboembolism, Pulmonary embolism, Deep vein thrombosis

## Abstract

**Background:**

Globally, sickle cell disease (SCD) is one of the most common haemoglobinopathy. Considered a public health problem, it leads to vessel occlusion, blood stasis and chronic activation of the coagulation system responsible for vaso-occlussive crises and venous thromboembolism (VTE) which may be fatal. Although contemporary observational studies suggest a relationship between SCD or sickle trait (SCT) and VTE, there is lack of a summary or meta-analysis data on this possible correlation. Hence, we propose to summarize the available evidence on the association between SCD, SCT and VTE including deep vein thrombosis (DVT) and pulmonary embolism (PE).

**Methods:**

We searched PubMed and Scopus to identify all cross-sectional, cohort and case-control studies reporting on the association between SCD or SCT and VTE, DVT or PE in adults or children from inception to April 25, 2017. For measuring association between SCD or SCT and VTE, DVT, or PE, a meta-analysis using the random-effects method was performed to pool weighted odds ratios (OR) of risk estimates.

**Results:**

From 313 records initially identified from bibliographic databases, 10 studies were eligible and therefore included the meta-analysis. SCD patients had significantly higher risk for VTE (pooled OR 4.4, 95%CI 2.6–7.5, *p* < 0.001), DVT (OR 1.1, 95% CI 1.1–1.2, *p* < 0.001) and PE (pooled OR 3.7, 95% CI 3.6–3.8, *p* < 0.001) as compared to non SCD-adults. A higher risk of VTE (OR 33.2, 95% CI 9.7–113.4, *p* < 0.001) and DVT (OR 30.7, 95% CI 1.6–578.2, *p* = 0.02) was found in pregnant or postpartum women with SCD as compared to their counterparts without SCD. Compared to adults with SCT, the risk of VTE was higher in adults with SCD (pooled OR 3.1, 95% CI 1.8–5.3, p < 0.001), and specifically in SCD pregnant or postpartum women (OR 20.3, 95% CI 4.1–102, *p* = 0.0003). The risk of PE was also higher in adults with SCD (OR 3.1, 95% CCI 1.7–5.9, *p* = 0.0004) as compared to those with SCT. The risk of VTE was higher in individuals with SCT compared to controls (pooled OR 1.7, 95% CI 1.3–2.2, *p* < 0.0001), but not in pregnant or postpartum women (OR 0.9, 95% CI 0.3–2.9, *p* = 0.863). Compared to controls, SCT was associated with a higher risk of PE (pooled OR 2.1, 95% CI 1.2–3.8, *p* = 0.012) but not of DVT (pooled OR 1.2, 95% CI 0.9–1.7, *p* = 0.157).

**Conclusion:**

Individuals with SCD, especially pregnant or postpartum women, might have a higher risk of VTE compared to the general population. SCT might also increases the risk of VTE. However, currently available data are not sufficient to allow a definite conclusion. Further larger studies are needed to provide a definitive conclusion on the association between SCD, SCT and VTE.

**Electronic supplementary material:**

The online version of this article (10.1186/s12959-018-0179-z) contains supplementary material, which is available to authorized users.

## Background

Sickle cell disease (SCD) is one of the most common severe monogenic disorders, affecting 20–25 million people globally [1, 2]. The clinical manifestations and complications of SCD result mainly from vaso-occlusion and/or hemolysis. When deoxygenated, hemoglobin S (HbS) polymerizes to form a fibrous network responsible for red cell rigidity, hemolysis, increased blood viscosity, poor microvascular blood flow and vessel occlusion [3]. Acute and chronic vessel occlusion could cause significant complications in various organs including the brain (strokes or silent brain infarcts), the kidneys (renal infarction with papillary necrosis or medullar fibrosis), the bones (aseptic osteonecrosis, pain crises), the spleen (spleen infarcts), the retina (retinopathy), the lungs (acute chest syndrome) or the male external genitalia (priapism) [3–5]. Some studies have also suggested an increased risk of venous thromboembolism (VTE) in patients with SCD or sickle cell trait (SCT) [6–9].

VTE which includes deep vein thrombosis (DVT) and its life-threatening complication, pulmonary embolism (PE), is a major contributor to global disease burden [10]. Thrombosis and subsequently embolism results from the interaction between blood stasis, vein wall injury and hypercoagulability (Virchow’s classic triad), playing on the background of genetic predispositions [11]. Vessel occlusion and blood stasis, as well as chronic activation of the coagulation system are plausible mechanisms to explain the possible increased risk of VTE in patients with SCD or SCT [12, 13].

While other thrombotic complications of SCD have been extensively studied, VTE has so far been overlooked as a major complication of SCD. Whether or not SCD and SCT are associated with VTE and the degree to which this possible association might influence our approach to the prevention of VTE in these specific populations, especially regarding anticoagulation remains unknown. Hence, we conducted this systematic review and meta-analysis to summarize existing evidence on the risk of VTE in people with SCD or SCT.

## Methods

This review is reported in accordance with the Preferred Reporting Items for Systematic reviews and Meta-Analyses (PRISMA) guidelines [14].

### Literature search

We performed a comprehensive and exhaustive search of Medline through PubMed and Scopus to identify all relevant articles published on VTE in individuals with SCD and SCT from inception to April 25, 2017. No language restriction was applied. We conceived and applied a search strategy for each bibliographic database based on the combination of relevant terms. Terms used for VTE included the following: “venous thromboembolism”, “pulmonary embolism”, and “deep venous thrombosis”. For SCD, the following and their variants were used: “sickle cell disease”, “sickle cell anaemia”, and “sickle cell trait”. We scanned the reference lists of all relevant reviews and all included studies to identify other potential eligible studies. Unpublished data were not sought for this review of observational studies because, unlike clinical trials which are now always registered, observational studies are usually not registered and unpublished data from such studies are hardly trackable.

### Selection of studies for inclusion in the review

We included cross-sectional, cohort and case-control studies reporting on the association between SCD or SCT and DVT or PE in male and female adults, pregnant and post-partum women as well as children. We excluded case series and studies lacking primary data. For studies published in more than one report (duplicates), the most comprehensive reporting the largest sample size was considered. SCD was defined as having the genotypes Hb SS, Hb SC, Hb SD or Hb S-thalassemia; SCT and normal control as having the genotypes Hb AS and Hb AA, respectively. PE was defined as the presence of a thrombus in the pulmonary vessels diagnosed by either CT-pulmonary angiography, magnetic resonance imaging (MRI), ventilation-perfusion lung (V/Q) scan or autopsy. DVT was defined as thrombosis of the deep venous circulation including limb, pelvic vessels, vena cava or cerebral vein diagnosed by either Doppler echography, venography, MRI or CT-Scan.

Two investigators independently screened all identified citations from the literature search results by title and abstract to identify articles for inclusion in the review (TNM and JJN). The full-texts of all citations that potentially met the inclusion criteria were then retrieved and assessed for final inclusion. All disagreements were resolved by discussion.

#### Data extraction and management

Two investigators (JJB and RT) independently extracted data from each included study using the data extraction form. Information extracted included: first author’s name, year of publication, period of data collection, country and corresponding WHO region, study design (cross-sectional, cohort or case-control), setting (hospital-based or community-based), sample size, mean or median age and age range, proportion of female participants, type of population (children, adults, pregnant women or postpartum women), comparators (SCD or SCT versus controls) and outcome (DVT, PE or both). Disagreements between investigators were resolved through consensus following a discussion.

#### Assessment of risk of bias in included studies

The methodological quality of included studies was evaluated using an adapted version of the Newcastle-Ottawa Scale (NOS) [15]. Two investigators (JJB and RT) independently assessed study quality, with disagreements resolved by discussion. A score of 0–3, 4–6, and 7–9 rated the risk of bias as high, moderate, and low.

#### Data synthesis and analysis

Data analyses used the *‘meta’* packages of the statistical software R (version 3.5.0, The R Foundation for statistical computing, Vienna, Austria). For measuring the association between SCD or SCT and VTE, DVT, or PE, a meta-analysis using the random-effects method of Der Simonian and Laird was performed to pool weighted odds ratios (OR) of risk estimates [16]. Raw data for measuring the association were extracted from each study and ORs were recalculated. Harbord test was done to assess the presence of publication bias [17]. A *p*-value < 0.10 was considered indicative of statistically significant publication bias. Heterogeneity across included studies was assessed using the χ^2^ test for heterogeneity with a 5% level of statistical significance [18], and by using the I^2^ statistic for which a value of 50% was considered to imply moderate heterogeneity [19]. Inter-rater agreements between investigators for study inclusion and methodological quality assessment were assessed using Kappa Cohen’s coefficient [20].

## Results

### The review process

Initially, 313 records were identified (79 in Scopus and 234 in PubMed). After elimination of duplicates and screening of title and abstracts, 19 records were selected and their full-texts downloaded. From the 19 full-texts scrutinized, 10 papers were found eligible and therefore included in the meta-analysis [21–30] (Additional file 1: Figure S1). The inter-rater agreement for study inclusion and data extraction between investigators was κ = 0.89 and 0.79 respectively.

### Characteristics of included studies

Additional file 2: Table S1 presents the characteristics of included studies. Seven studies had low risk of bias and three had moderate risk of bias. The included studies were published from 2006 to 2017. Eight studies were conducted in general adult populations [21–27], one in pregnant women only [28] and two in both pregnant and postpartum women [29, 30]. In the seven studies carried out in the general adult populations [21–27], the proportion of female varied from 25.0 to 100%. Seven studies were from the USA [21–25, 29, 30], one from the UK [26], one from Nigeria [27], and one from Brazil [28].

### Sickle cell disease versus control

**Fig. 1:**
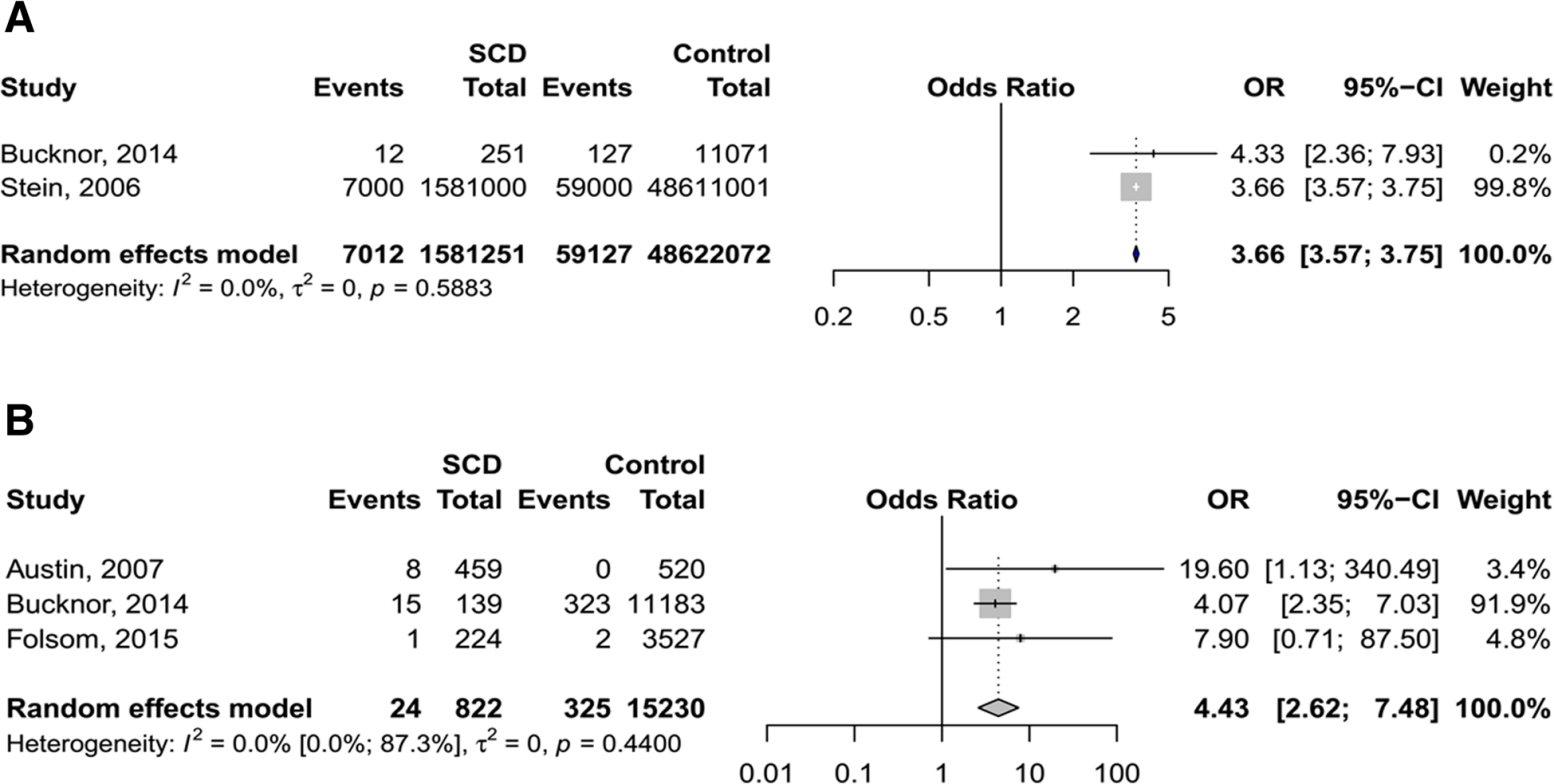
**a** Risk of PE in sickle cell disease patients vs controls without sickle cell disease. **b** Risk of VTE in sickle cell disease patients vs controls without sickle cell disease

Data from three studies showed that SCD patients had significantly higher risk for VTE as compared to non SCD-individuals (pooled OR 4.4, 95%CI 2.6–7.9, *p* < 0.001; Fig. 1) [23–25]. The risk of PE was also higher in individuals with SCD (pooled OR 3.7, 95% CI 3.6–3.8, p < 0.001) as demonstrated in two studies (Fig. 1) [21, 24]. One study showed a slightly increased risk of DVT associated with SCD (OR 1.1, 95% CI 1.1–1.2, *p* < 0.001; Table 1) [21].

**Table 1:** Summary statistics

Outcome	N studies	Population	Odds ratio (95%CI)	*P* value	H (95%CI)	I^2^ (95%CI)	P heterogeneity	P Harbord test
SCD versus Control								
VTE	3	Adults	4.43 (2.62–7.48)	<.0001	1.0 (1.0–2.8)	0.0 (0.0–87.3)	.440	.467
DVT	1	Adults	1.12 (1.09–1.15)	<.0001	NA	NA	NA	NA
PE	2	Adults	3.66 (3.57–3.75)	<.0001	1.0	0.0	0.588	NA
VTE	1	Pregnant and PP women	33.16 (9.70–113.37)	<.0001	NA	NA	NA	NA
DVT	1	Pregnant women	30.66 (1.63–578.15)	.022	NA	NA	NA	NA
SCD versus SCT								
VTE	3	Adults	3.09 (1.79–5.33)	<.0001	1.0 (1.0–2.2)	0.0 (0.0–79.8)	.598	.524
PE	1	Adults	3.14 (1.67–5.92)	.0004	NA	NA	NA	NA
VTE	1	Pregnant and PP women	20.34 (4.05–102.05)	.0003	NA	NA	NA	NA
SCT versus Control								
VTE	4	Adults	1.71 (1.34–2.18)	<.0001	1.0 (1.0–2.5)	0.0 (0.0–83.5)	.427	.238
DVT	3	Adults	1.23 (0.92–1.65)	.157	1.0 (1.0–1.5)	0.0 (0.0–55.7)	.791	.810
PE	3	Adults	2.12 (1.18–3.80)	.012	2.3 (1.3–4.0)	80.6 (39.0–93.8)	.006	.405
VTE	2	Pregnant and PP women	0.90 (0.28–2.89)	.863	1.48	47.5	0.168	NA

*CI* confidence interval, *DVT* deep venous thrombosis, *VTE* venous thromboembolism, *PE* pulmonary embolism, *PP* postpartum, *SCD*, sickle cell disease, *SCT* sickle cell trait, *NA* not applicable

Two studies reported higher risk of VTE (OR 33.7, 95% CI 9.7–113.4, p < 0.001) and DVT (OR 30.7, 95% CI 1.6–578.2, *p* = 0.02) in pregnant or postpartum women with SCD as compared to their counterparts without SCD (Table 1) [28, 30].

### Sickle cell disease versus sickle cell trait

**Fig. 2:**
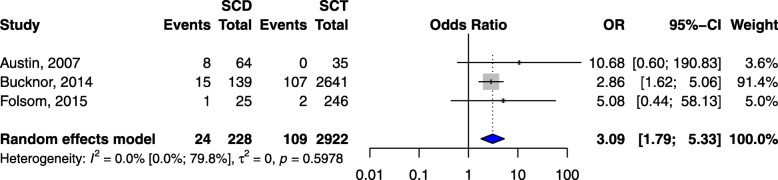
Risk of VTE in sickle cell disease patients vs individuals with sickle cell trait

Compared to adults with SCT, the risk of VTE was higher in adults with SCD (pooled OR 3.1, 95% CI 1.8–5.3, *p* < 0.001) as shown in 3 studies (Fig. 2) [23–25], and in SCD pregnant or postpartum women (OR 20.3, 95% CI 4.1–102.1, *p* = 0.0003, 1 study, Table 1) [30] as compared to their counterparts with SCT. The risk of PE was also higher in adults with SCD (OR 3.1, 95% CCI 1.7–5.9, *p* = 0.0004) as found in one study (Table 1) [24]. No study reported DVT as the outcome.

### Sickle cell trait versus control

**Fig. 3:**
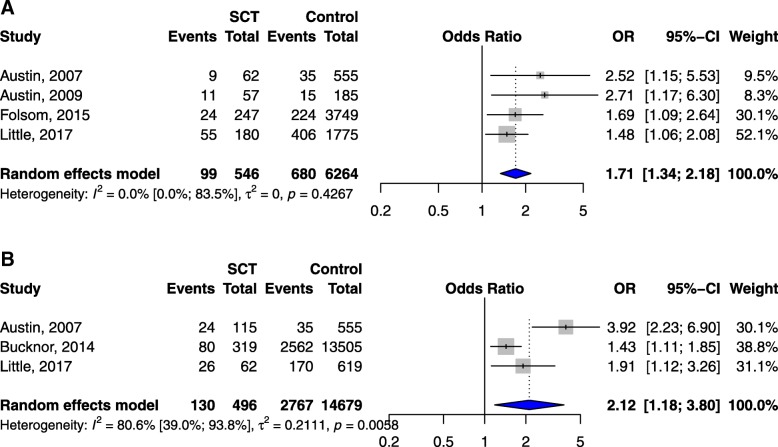
**a** Risk of VTE in individuals with sickle cell trait vs controls. **b** Risk of PE in individuals with sickle cell trait vs controls

Four studies showed that the risk of VTE was higher in individuals with SCT compared to controls (pooled OR 1.7, 95% CI 1.3–2.2, *p* < 0.0001, Fig. 3) [22, 23, 25, 26], but not in pregnant or postpartum women (OR 0.9, 95% CI 0.3–2.9, *p* = 0.863, Table 1 and Additional file 3: Figure S2) as reported in two studies [29, 30]. Higher risk of PE was also associated with SCT (pooled OR 2.1, 95% CI 1.2–3.8, *p* = 0.012, Fig. 3) [23, 24, 26]. However, SCT did not increase the risk of DVT (pooled OR 1.2, 95% CI 0.9–1.7, *p* = 0.157, Table 1 and Additional file 4: Figure S3) in 3 studies [23, 26, 27].

## Discussion

This systematic review and meta-analysis is the first to summarize available evidence on the association between SCD or SCT and VTE, DVT and PE. We found that (1) SCD was associated with a significantly higher risk of VTE and specifically DVT and PE compared to non-SCD controls; (2) the risk of VTE and PE in adults with SCD and of VTE in SCD pregnant/postpartum women was higher than in their counterparts with SCT; (3) compared to controls, individuals with SCT had a higher risk of VTE and PE but not DVT; (4) SCT was not associated with a higher risk of VTE in women during pregnancy or postpartum.

SCD-specific mechanisms causing hypercoagulability are believed to contribute significantly to the higher risk of VTE in the SCD population compared to the general population. Alterations of sickled red cells structure lead to haemolysis, release of prothrombotic substances such as phosphatidylserines and nitric oxide depletion [31, 32], causing platelets and coagulation cascade activation, impaired fibrinolysis, vaso-constriction, reduced blood flow and ischaemic vascular injury [32–35]. All these alterations ultimately result in hypercoagulability, endothelial dysfunction and blood stasis (Virchow’s classic triad) causing thrombosis and subsequently embolism [13, 35, 36]. Furthermore, several thrombophilic abnormalities are associated with SCD. Antiphospholipid antibodies such as lupus anticoagulant, anticardiolipin and antiphosphatidylserine antibodies are highly prevalent in SCD patients, and are associated with thrombotic complications including stroke [13, 37]. The production of these auto-antibodies is thought to result from structural changes in sickled red cell membrane [37]. Genetic deficiency in Protein C and S is a known risk factor for VTE. Low circulating levels of Protein C and S found in SCD patients and which are due to chronic consumption in the context of continuous activation of coagulation cascade on the surface of sickled red blood cells, may also play a role in the thrombogenic proclivity of these patients [13, 37, 38].

Surgical splenectomy which is frequently performed in SCD patients is also potential risk factor for VTE in SCD [13]. A study which evaluated the association of surgical splenectomy with VTE in patients with sickle variant genotypes found that 27% of patients with VTE had a history of splenectomy compared to 6% without VTE, suggesting that splenic dysfunction might increase VTE risk in SCD [8]. Furthermore, few studies have revealed surgical splenectomy as a significant risk factor for VTE in the general population [39] and in hereditary haemolytic conditions such as β-thalassemia and hereditary spherocytosis [40, 41]. Few pathophysiologic mechanisms underlying the increased post-splenectomy thrombotic risk have been hypothesized, including chronic intravascular haemolysis, reduced clearance of abnormal red blood cells and hypercoagulability [36, 42, 43]. Functional asplenia secondary to splenic auto-infarction is a very common and early complication in SCD which occurs in about 90% of homozygote SS infants by age 1 and a large proportion of patients with SC genotype by mid-childhood [44]. Functional asplenia may also enhance hypercoagulability and consequentially increase the risk of VTE in SCD. However there is no available epidemiological data to support this association as it is the case for surgical splenectomy [13].

We found an increased risk of VTE in pregnant women with SCD compared to those without SCD. However, the very broad 95% CI associated to the odd ratio (~ 30) suggests a significant uncertainty of this association. Pregnancy itself is a well-recognised risk factor for VTE due to high levels of oestrogen and progesterone which are prothrombotic hormones [45, 46]. Our findings suggest that the combination of SCD and pregnancy has a multiplicative prothrombotic effect which is by far greater than the effect of each of them separately. One study showed that the prevalence of VTE was 3.5-fold greater among SCD pregnant women with a subset of complications such as vaso-occlusive crisis, acute chest syndrome and pneumonia compared to those without these complications [9]. Altogether, these data highlight the need for effective thromboprophylaxis in SCD women during pregnancy, especially those with concurrent thrombotic complications such as vaso-occlusive crisis or acute chest syndrome. Furthermore, as the majority of VTE seems to occur in the postpartum period [29], special attention should be given during this period and non-pharmacological measures such as mobilization should be encouraged early in the hospital and after discharge.

We found a higher risk of VTE and specifically PE associated with SCT in the general population. Our data suggest that not only SCD but also SCT are prothrombotic states. Interestingly, there is growing evidence underscoring a greater risk of VTE in individuals of African descent compared to Caucasians [47–50]. Considering the high prevalence of SCT in populations of African descent such as African Americans (7% to 10%) or sub-Saharan Africans (up to 30%) [51–53], it is plausible that the prothrombotic tendency of SCT may contribute to the higher incidence of VTE in individuals of African descent. However, we did not find an association between SCT and increased risk of VTE in pregnant or postpartum women. It is possible that there may be confounding factors that can drown out a possible association. Indeed, of the two studies included in this analysis, one had an age difference between the two groups compared while in the other, the compared groups differed in the distribution of the presence of diabetes at delivery. Furthermore, we observed no association between SCT and DVT, a finding in contrast with the potential link between SCT and PE. As mentioned above, it is possible that some confounding factors as well the small numbers of cases of SCT and controls hid a possible association between SCT and DVT.

Our finding of a higher risk of VTE in individuals with SCD compared to those without SCD underscore the need for a specific approach to the management of VTE in this population. Unfortunately there is no available evidence to inform anticoagulation practices in these patients. Therefore, our findings stress the need for studies to investigate anticoagulation modalities and other therapies such as hydroxyurea in the prevention and treatment of VTE in individuals with SCD including pregnant women. Before such evidence become available, it is important for clinicians to tailor their decisions regarding thromboprophylaxis and treatment to the specificities of SCD patients [13]. Hospitalized patients with SCD might benefit from thromboprophylaxis at a younger age as their risk of VTE is markedly higher than that of non-SCD individuals of the same age. While prophylactic anticoagulation is sometimes eluded in patients with low haemoglobin levels, clinicians should remember that SCD patients have lower baseline haemoglobin levels, and thus avoid an overestimation of the bleeding risk in these patients. Furthermore, the algorithms used for the diagnosis and treatment of DVT and PE should be interpreted carefully for patients with SCD. For instance, a pivotal marker like D-dimer is not reliable in these patients [13].

Our study has some limitations, mainly the limited number of studies found on the topic and included in the review. This demonstrates that VTE is still a neglected issue in the SCD population. As a consequence of the limited number of studies included and data analysed, it was not possible to assess the influence on our estimates of potential confounders such as the classic risk factors of VTE. Much more, no stratified analysis was done pertaining to the different SCD genotypes which have been shown to affect the occurrence of complications. For instance, a higher prevalence of PE was found in patients with Hb SC and Sβ + thalassemia compared to those with Hb SS [54]. Prospective studies on large cohorts of both SCD and SCT, specifically in sub-Saharan where most of patients and sickle cell carriers live are desirable. Highly elevated odds ratio found when measuring the association between DVT, VTE and exposure to SCD and SCT among pregnant women should be interpreted with caution when looking at the broad confidence interval found; the association may be lower or higher than actually presented. This large confidence interval may be explained by lack of power highlighting the need of more research in this field.

## Conclusion

This review indicates that individuals with SCD, especially women during pregnancy and the postpartum period, might have a higher risk of VTE compared to the general population. SCT might also increase the risk of VTE. However, currently available data are not sufficient to allow a definite conclusion. Further larger studies are needed to provide a definitive conclusion on the association between SCD, SCT and VTE, in order to potentially inform specific approaches to the management of VTE in the SCD population.

## Additional files

Additional file 1: **Figure S1.** PRISMA Flow Chart (TIF 25 kb)

Additional file 2: **Table S1.** Characteristics of included studies (DOCX 25 kb)

Additional file 3: **Figure S2.** Risk of VTE in pregnant or postpartum women with sickle cell trait vs controls. (PDF 5 kb)

Additional file 4: **Figure S3.** Risk of DVT in individuals with sickle cell trait vs controls (PDF 5 kb)

## Abbreviations

DVT: Deep vein thrombosis
PE: Pulmonary embolism
SCD: Sickle cell disease
SCT: Sickle cell trait
VTE: Venous thromboembolism
