# Supplementary figures and images for: Sickle cell disease, sickle trait and the risk for venous thromboembolism: a systematic review and meta-analysis
Source: Thromb J. 2018 Oct 4;16:27. doi: 10.1186/s12959-018-0179-z (PMC6171302; doi:10.1186/s12959-018-0179-z)

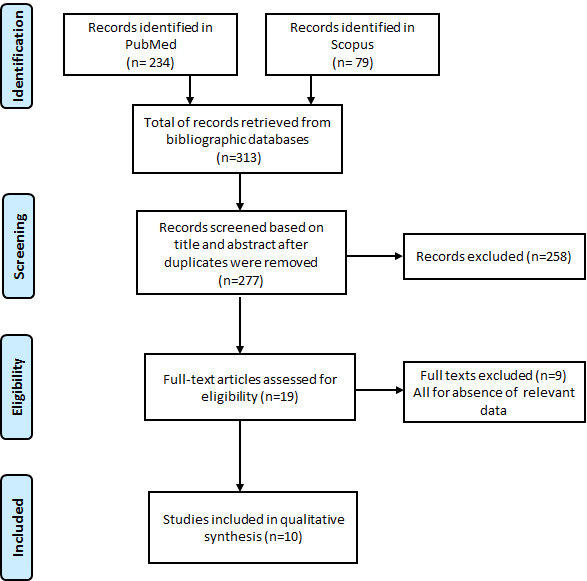

Supplement: Supplementary file 1 — Figure S1. PRISMA Flow Chart (TIF 25 kb) [file 12959_2018_179_MOESM1_ESM.tif]

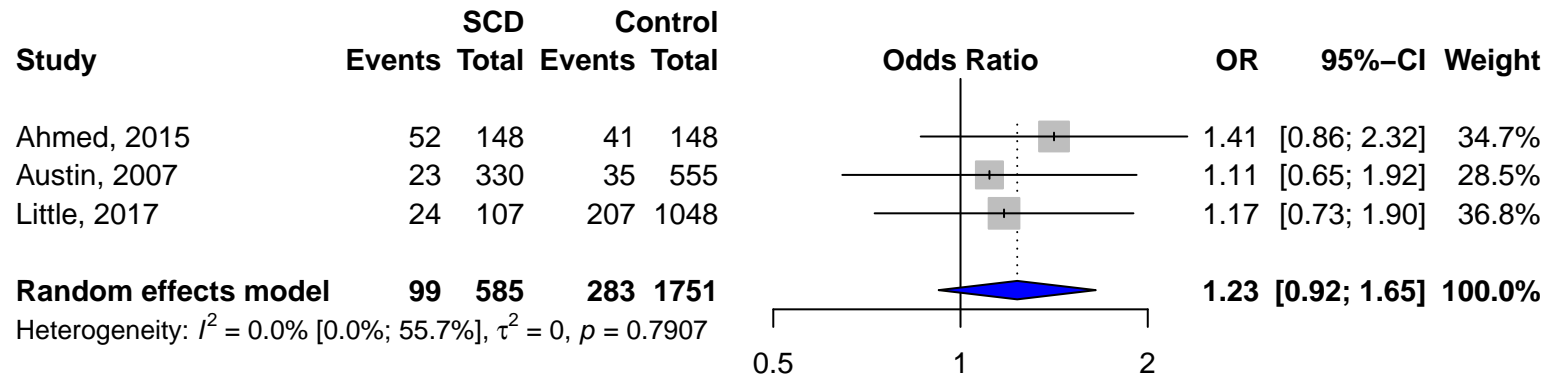

Supplement: Supplementary file 3 — Figure S2. Risk of VTE in pregnant or postpartum women with sickle cell trait vs controls. (PDF 5 kb) [file 12959_2018_179_MOESM3_ESM.pdf]

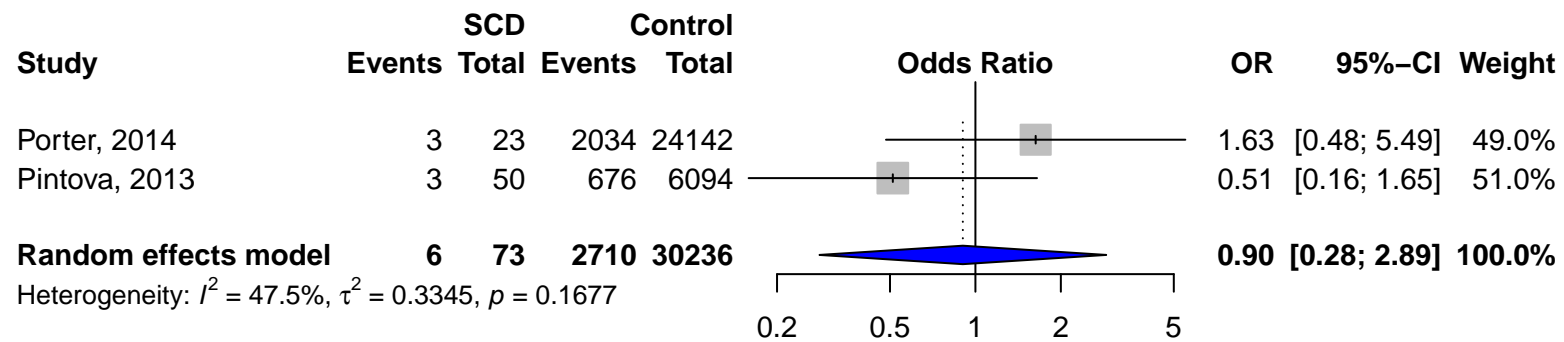

Supplement: Supplementary file 4 — Figure S3. Risk of DVT in individuals with sickle cell trait vs controls (PDF 5 kb) [file 12959_2018_179_MOESM4_ESM.pdf]
