# Supplementary material for: Sickle cell disease, sickle trait and the risk for venous thromboembolism: a systematic review and meta-analysis
Source: Thromb J. 2018 Oct 4;16:27. doi: 10.1186/s12959-018-0179-z (PMC6171302; doi:10.1186/s12959-018-0179-z)
Supplement: Supplementary file 2 — Table S1. Characteristics of included studies (DOCX 25 kb) [file 12959_2018_179_MOESM2_ESM.docx]

**Table S1 Characteristics of included studies**

| **Study** | **Population** | **Outcome** | **Comparison** | **Exposed with disease** | **Non exposed with disease** | **Exposed without disease** | **Non exposed without disease** | **%Female** | **Confusion** | **Age distribution, years** | **No difference between cases and control** | **Higher in cases or exposed** | **Higher in non-cases or non-exposed** | **Study design** | **Country** | **Timing of data collection** | **WHO Region** | **Sampling method** | **Study site** | **Period of inclusion of participants** | **Inclusion criteria of participants** |
| --- | --- | --- | --- | --- | --- | --- | --- | --- | --- | --- | --- | --- | --- | --- | --- | --- | --- | --- | --- | --- | --- |
| Ahmed, 2015 | Adults | DVT | SCT versus Control | 52 | 96 | 41 | 107 |  | NR | Median (range)  Cases: 54 (33-84)  Control: 52 (30_82) | age, sex |  |  | Case control | Nigeria | Retrospectively | Africa | Consecutive sampling | Hospital-based | 1996-2010 | DVT patients with characteristic clinical features including lower limb edema, warmth and/or tenderness associated with ultrasonographically demonstrated intravenous thrombosis with or without associated PE |
| Austin, 2007 | Adults | PE | SCT versus Control | 24 | 91 | 35 | 520 | 41.4 | NR | Median (1^st^ – 3^rd^ quartile)  Cases: 48 (38-56)  Control: 49 (38-58) | sex, hypertension, obesity, factor V Leinden, Prothrombin mutation | age, diabetes, smoking, Family history of VTE | exercise | Case control | USA | Prospectively | Americas | Consecutive sampling | Hospital-based | 1997-2005 | Cases: aged 18 to 70 with a recently diagnosed first or recurrent episode of a DVT and/or PE. Controls : matched for age, sex and race |
| Austin, 2007 | Adults | VTE | SCT versus Control | 9 | 53 | 35 | 520 | 41.4 | NR | Median (1^st^ – 3^rd^ quartile)  Cases: 48 (38-56)  Control: 49 (38-58) | sex, hypertension, obesity, factor V Leinden, Prothrombin mutation | age, diabetes, smoking, Family history of VTE | exercise | Case control | USA | Prospectively | Americas | Consecutive sampling | Hospital-based | 1997-2005 | Cases: aged 18 to 70 with a recently diagnosed first or recurrent episode of a DVT and/or PE. Controls : matched for age, sex and race |
| Austin, 2007 | Adults | VTE | SCD versus Control | 8 | 451 | 0 | 520 | 41.4 | NR | Median (1^st^ – 3^rd^ quartile)  Cases: 48 (38-56)  Control: 49 (38-58) | age, sex, hypertension, obesity, factor V Leinden, Prothrombin mutation | diabetes, smoking, Family history of VTE | exercise | Case control | USA | Prospectively | Americas | Consecutive sampling | Hospital-based | 1997-2005 | Cases: aged 18 to 70 with a recently diagnosed first or recurrent episode of a DVT and/or PE. Controls : matched for age, sex and race |
| Austin, 2007 | Adults | VTE | SCD versus SCT | 8 | 56 | 0 | 35 | 41.4 | NR | Median (1^st^ – 3^rd^ quartile)  Cases: 48 (38-56)  Control: 49 (38-58) | age, sex, hypertension, obesity, factor V Leinden, Prothrombin mutation | diabetes, smoking, Family history of VTE | exercise | Case control | USA | Prospectively | Americas | Consecutive sampling | Hospital-based | 1997-2005 | Cases: aged 18 to 70 with a recently diagnosed first or recurrent episode of a DVT and/or PE. Controls : matched for age, sex and race |
| Austin, 2007 | Adults | DVT | SCT versus Control | 23 | 307 | 35 | 520 | 41.4 | NR | Median (1^st^ – 3^rd^ quartile)  Cases: 48 (38-56)  Control: 49 (38-58) | sex, hypertension, obesity, factor V Leinden, Prothrombin mutation | age, diabetes, smoking, Family history of VTE | exercise | Case control | USA | Prospectively | Americas | Consecutive sampling | Hospital-based | 1997-2005 | Cases: aged 18 to 70 with a recently diagnosed first or recurrent episode of a DVT and/or PE. Controls : matched for age, sex and race |
| Austin, 2009 | Adults | VTE | SCT versus Control | 11 | 46 | 15 | 170 | 100 | Yes | Mean  Cases: 36  Control: 38 | age, household income | Hormonal contraception |  | Case control | USA | Prospectively | Americas | Not random or consecutive sampling | Hospital-based |  | Cases: African American women aged 18-49 years with a recently diagnosed first episode of DVT and/or PE without an underlying provocation for their VTE. Controls matched for age. |
| Bucknor, 2014 | Adults | PE | SCT versus Control | 80 | 239 | 2562 | 10943 | 68.9 | No | Median, Mean ± Standard deviation  SCD: 31; 35.4 ± 14.5  SCT: 32; 35.9 ± 0.1  Control: 36; 37.8 ± 13.6 | age, diabetes mellitus |  | Female sex, obesity, hyperlipidemia | Cohort | USA | Retrospectively | Americas | Consecutive sampling | Hospital-based | 1995-2008 | African Americans aged at least 18 years of age |
| Bucknor, 2014 | Adults | PE | SCD versus Control | 12 | 239 | 127 | 10944 | 68.9 | No | Median, Mean ± Standard deviation  SCD: 31; 35.4 ± 14.5  SCT: 32; 35.9 ± 0.1  Control: 36; 37.8 ± 13.6 | age, diabetes mellitus |  | Female sex, obesity, hyperlipidemia | Cohort | USA | Retrospectively | Americas | Consecutive sampling | Hospital-based | 1995-2008 | African Americans aged at least 18 years of age |
| Bucknor, 2014 | Adults | PE | SCD versus SCT | 12 | 80 | 127 | 2662 | 68.9 | No | Median, Mean ± Standard deviation  SCD: 31; 35.4 ± 14.5  SCT: 32; 35.9 ± 0.1  Control: 36; 37.8 ± 13.6 | age, diabetes mellitus |  | Female sex, obesity, hyperlipidemia | Cohort | USA | Retrospectively | Americas | Consecutive sampling | Hospital-based | 1995-2008 | African Americans aged at least 18 years of age |
| Bucknor, 2014 | Adults | VTE | SCD versus Control | 15 | 124 | 323 | 10860 | 68.9 | No | Median, Mean ± Standard deviation  SCD: 31; 35.4 ± 14.5  SCT: 32; 35.9 ± 0.1  Control: 36; 37.8 ± 13.6 | age, diabetes mellitus |  | Female sex, obesity, hyperlipidemia | Cohort | USA | Retrospectively | Americas | Consecutive sampling | Hospital-based | 1995-2008 | African Americans aged at least 18 years of age |
| Bucknor, 2014 | Adults | VTE | SCD versus SCT | 15 | 124 | 107 | 2534 | 68.9 | No | Median, Mean ± Standard deviation  SCD: 31; 35.4 ± 14.5  SCT: 32; 35.9 ± 0.1  Control: 36; 37.8 ± 13.6 | age, diabetes mellitus |  | Female sex, obesity, hyperlipidemia | Cohort | USA | Retrospectively | Americas | Consecutive sampling | Hospital-based | 1995-2008 | African Americans aged at least 18 years of age |
| Costa, 2014 | Pregnant women | DVT | SCD versus Control | 4 | 0 | 56 | 192 | 100 | NR | Not reported | age, parity, newborn sex |  |  | Cohort | Brazil | Prospectively | Americas | Consecutive sampling | Hospital-based | 2009-2011 | Cases: pregnant women before completing 20 weeks of gestation. Controls: matched for maternal age, maternal parity and newborn’s sex |
| Folsom, 2015 | Adults | VTE | SCD versus Control | 1 | 223 | 2 | 3525 | 38.3 | NR | Mean ± Standard deviation  Cases: 53.8 ± 5.9  Control: 53.5 ± 5.8 |  |  |  | Cohort | USA | Prospectively | Americas | Not random or consecutive sampling | Population/Community-based | 1987-1989 | African-American men and women aged 45 to 64 years |
| Folsom, 2015 | Adults | VTE | SCD versus SCT | 1 | 24 | 2 | 244 | 38.3 | NR | Mean ± Standard deviation  Cases: 53.8 ± 5.9  Control: 53.5 ± 5.8 |  |  |  | Cohort | USA | Prospectively | Americas | Not random or consecutive sampling | Population/Community-based | 1987-1989 | African-American men and women aged 45 to 64 years |
| Folsom, 2015 | Adults | VTE | SCT versus Control | 24 | 223 | 224 | 3525 | 38.3 | Yes | Mean ± Standard deviation  Cases: 53.8 ± 5.9  Control: 53.5 ± 5.8 | age, sex, hormonal contraception, BMI, Activated partial thromboplastin time, Fibrinogen, Antithrombin III | Factor VII, Factor VIII, von Willebrand factor, Protein C |  | Cohort | USA | Prospectively | Americas | Not random or consecutive sampling | Population/Community-based | 1987-1989 | African-American men and women aged 45 to 64 years |
| Little, 2017 | Adults | DVT | SCT versus Control | 24 | 83 | 207 | 841 | 82.5 | No | Median (1^st^-3^rd^ quartiles)  Cases: 32 (35-41)  Control: 28 (22-34) | age, sex, ethnicity, smoking | BMI, pregnancy |  | Case control | UK | Retrospectively | Europe | Consecutive sampling | Hospital-based | 1988-2013 | Screened for sickle cell between age 18 and 75 years with the first occurrence of VTE |
| Little, 2017 | Adults | PE | SCT versus Control | 26 | 36 | 170 | 449 | 82.5 | No | Median (1^st^-3^rd^ quartiles)  Cases: 32 (35-41)  Control: 28 (22-34) | age, sex, ethnicity, smoking | BMI, pregnancy |  | Case control | UK | Retrospectively | Europe | Consecutive sampling | Hospital-based | 1988-2013 | Screened for sickle cell between age 18 and 75 years with the first occurrence of VTE |
| Little, 2017 | Adults | VTE | SCT versus Control | 55 | 125 | 406 | 1369 | 82.5 | No | Median (1^st^-3^rd^ quartiles)  Cases: 32 (35-41)  Control: 28 (22-34) | age, sex, ethnicity, smoking | BMI, pregnancy |  | Case control | UK | Retrospectively | Europe | Consecutive sampling | Hospital-based | 1988-2013 | Screened for sickle cell between age 18 and 75 years with the first occurrence of VTE |
| Pintova, 2013 | Pregnancy and Post-Partum | VTE | SCT versus Control | 3 | 47 | 676 | 5418 | 100 | NR | Mean ± Standard deviation  Cases: 32.2 ± 7.8  Control: 27.6 ± 6.4 | Race black | age |  | Cross sectional | USA | Retrospectively | Americas | Consecutive sampling | Hospital-based | 1998-2008 | All pregnant/postpartum non-Hispanic women who delivered |
| Porter, 2014 | Pregnancy and Post-Partum | VTE | SCT versus Control | 3 | 20 | 2034 | 22108 | 100 | NR | Mean ± Standard deviation  SCD: 21.8 ± 5.7  SCT: 21.9 ± 5.6  Control: 21.9 ± 5.6 | age, BMI, education, marital status, smoking, alcohol, drug, hypertension at delivery, nulliparous | Diabetes mellitus at delivery |  | Cohort | USA | Retrospectively | Americas | Consecutive sampling | Hospital-based | 1991-2006 | African American women with more than one prenatal visit |
| Porter, 2014 | Pregnancy and Post-Partum | VTE | SCD versus Control | 3 | 20 | 100 | 22108 | 100 | NR | Mean ± Standard deviation  SCD: 21.8 ± 5.7  SCT: 21.9 ± 5.6  Control: 21.9 ± 5.6 | age, BMI, education, marital status, smoking, alcohol, drug, hypertension at delivery, nulliparous | Diabetes mellitus at delivery |  | Cohort | USA | Retrospectively | Americas | Consecutive sampling | Hospital-based | 1991-2006 | African American women with more than one prenatal visit |
| Porter, 2014 | Pregnancy and Post-Partum | VTE | SCD versus SCT | 3 | 3 | 100 | 2034 | 100 | NR | Mean ± Standard deviation  SCD: 21.8 ± 5.7  SCT: 21.9 ± 5.6  Control: 21.9 ± 5.6 | age, BMI, education, marital status, smoking, alcohol, drug, hypertension at delivery, nulliparous | Diabetes mellitus at delivery |  | Cohort | USA | Retrospectively | Americas | Consecutive sampling | Hospital-based | 1991-2006 | African American women with more than one prenatal visit |
| Stein, 2006 | Adults | DVT | SCD versus Control | 7000 | 193000 | 1574000 | 48418000 |  | NR | Not reported |  |  | age | Cross sectional | USA | Retrospectively | Americas | Consecutive sampling | Hospital-based | 1979-2003 | Patients included with sickle cell disease and trait |
| Stein, 2006 | Adults | PE | SCD versus Control | 7000 | 59000 | 1574000 | 48611001 |  | NR | Not reported | age |  |  | Cross sectional | USA | Retrospectively | Americas | Consecutive sampling | Hospital-based | 1979-2003 | Patients included with sickle cell disease and trait |
